# Awareness of and attitudes towards public involvement in research on ageing and health among older people in Sweden

**DOI:** 10.1371/journal.pone.0269993

**Published:** 2022-06-21

**Authors:** Joakim Frögren, Steven M. Schmidt, Maya Kylén, Oskar Jonsson, Björn Slaug, Susanne Iwarsson

**Affiliations:** Department of Health Sciences, Lund University, Lund, Sweden; Curtin University, AUSTRALIA

## Abstract

**Background:**

While the importance of involving older people in research is increasingly acknowledged, quantitative studies exploring the perspectives of larger samples of older people who take an active role in research on ageing and health are scarce. The aim of this study was to investigate the awareness of and attitudes towards public involvement in research on ageing and health among older people in Sweden.

**Materials and methods:**

Data derived from a survey (N = 881) of people aged 60 years or older in Sweden. Demographics, self-rated health, and attitudes were analysed using descriptive statistics. Awareness of and previous active involvement in research were analysed using chi-square tests and Mann Whitney tests. Factors associated with willingness to be actively involved in research were determined by logistic regressions.

**Results:**

Of the 26% who responded (N = 881), 39% (n = 343) were aware that they could be actively involved in research. Awareness and previous active involvement in research were significantly associated with a higher level of education. Public involvement was believed to enhance research communication and enable valuable contributions related to ageing. The proportion of respondents who were willing to be actively involved in research was significantly higher for respondents with previous experience and a higher level of education.

**Conclusions:**

Engaging older people in Sweden in research targeting active involvement in research presents a challenge. The study shows an over-representation of people with higher education, who tend to be more aware, have previous experience, and are more willing to be involved in research with public involvement. This implies a risk that groups with lower education are not represented, and that knowledge co-produced with mostly highly educated groups will lead to a biased picture. Further studies are needed to understand how an increased awareness of research and willingness to participate can be achieved.

**IRRID:** RR2-10.2196/17759.

## Introduction

Public involvement in research is defined as “research being carried out ‘with’ or ‘by’ members of the public rather than ‘to,’ ‘about’ or ‘for’ them” [1]. This could be, for example, acting as an advisor in a steering group for a research project, being involved in developing or commenting on research material, or being involved in collecting data. Members of the public who are involved in research tend to come from specific populations, such as certain patient groups, service users, or people who share a common experience or demographic characteristics [2]. However, little is known about the awareness of and attitudes towards public involvement in research from a population perspective.

A plethora of terms have been used to describe the phenomenon of involving members of the public in research, such as ‘patient and public involvement’ (PPI), ‘patient-driven research’, ‘user involvement’, ‘community-based participatory research’ (CBPR), and ‘consumer participation’ [3, 4]. This is partly because different populations are referred to in different cases, but it also relates to traditions in the research field and approaches to research. In this study, we employ the terms ‘public involvement’ and ‘active involvement’ interchangeably. However, occasionally, ‘user involvement’ is used because this term was employed for the research program [5] from which the present study derived, and the term includes a greater breadth of user categories than just members of the public.

### Arguments for public involvement in research

Historically, public involvement in research has its origins in fields such as action research, adult education, international development, and critical sociology [6]. The emergence of public involvement in research has taken place based on a pathos of justice and a desire to protect and empower various marginalized groups [6, 7]. Regarding older people, it has been pointed out that an ethical reason for actively involving the population in research relates to “their rights as citizens to influence decisions which could affect their lives in the long term, and the need to further social justice and human rights in the context of power differences between people” [4, p. 2]. One reason why this population segment needs increased power or empowerment is related to the adverse effects of ageism and the marginalization of older people in today’s society [8, 9], which has been observed during the COVID-19 pandemic [10]. Another reason is the need to better understand the diverse needs and attitudes of this population segment. Older people are far from a homogeneous collective and display major and increasing differences in terms of past experiences, resources, and expectations.

In addition, public involvement in university-based research is rooted in societal developments where universities, in their dual role as public authorities and places for the production of knowledge and innovation have been given greater demands to serve their clients (citizens, research funders, etc.) and become more efficient [11, 12]. Today, universities are expected, based on their role as public authorities, to reflect society at large in terms of representativeness and interests. Moreover, the knowledge produced at universities is also expected to be applicable and useful outside of the university walls, i.e., *socially robust knowledge* [11]. Developments in the manufacturing industry that have moved away from mass production to the development of products and services that are to a greater extent based on users’ interests and needs have also had an influence on university-based research [12]. Thus, it is from this perspective that the involvement of members of the public in research should be understood.

Accordingly, there are two main arguments for public involvement in research. The first is about ethics and democracy: those affected by the research–directly or indirectly–should have the right to have an opinion on how it is being conducted. The second is about the relevance, quality, validity, and utilization of the research that is produced, that is, assuring that the co-created knowledge “works” and is applicable to the phenomenon it intends to describe or the problem it intends to solve. For these reasons, public involvement in research has strong political support and is frequently stated as a requirement for obtaining funding for research projects [13]. Still, a recent systematic review regarding involvement in research and health care in Europe indicated that public involvement is not yet firmly anchored or sufficiently formalized and that this might be due to a lack of infrastructure, guidance, and support [14].

### Earlier research on awareness of and attitudes towards public involvement in research

In this paper, awareness is defined as having knowledge of and understanding that something is happening or exists [15]. We have not been able to identify a single study that investigates how aware people are that research approaches with public involvement exist. Thus, to what extent the population in general, and older people in particular, are aware of such research approaches is largely unknown. In this paper, attitude is defined as the way that one thinks and feels about something or someone [16], while willingness is defined as being inclined or favourably disposed towards something [17]. In terms of older people’s active involvement in research, a systematic review [18] indicates that older people have a positive attitude towards such involvement, especially if the research results can contribute to a real change in practice in the form of, for example, improved services or policies.

However, previous research suggests that there are health-related factors that affect the willingness to be involved in research. A review of the literature on methods for involving older people in health research [19] indicated that their perceived health conditions or self-rated health can affect their ability or willingness to get involved. However, the same study showed that there are strategies that can promote the effective involvement of older adults with compromised health in research, such as thoughtful choice of location, use of accessible communication, building good relationships, and flexible approaches. That negative effect of health conditions on the willingness to be actively involved has also been confirmed by other studies, such as Berge et al.’s [20] and Holroyd-Leduc et al.’s [21] studies of frail older people’s experiences of involvement in research and Davies et al.’s [22] study about engaging very old people in research.

Existing research on attitudes towards public involvement among older people cites both benefits and challenges, for example, that involvement strengthens self-esteem [23] and results in learning opportunities [24]. Such research efforts have sometimes been criticized as tokenistic [25], creating demanding workloads, difficult relationships, and dissatisfaction with the level of involvement [26]. Most of these evaluative studies are small qualitative studies or systematic reviews based on such studies, with an emphasis on individual retrospective evaluations of experiences of being actively involved in research.

Larger quantitative studies exploring the perspectives of older people regarding their awareness, attitudes, and willingness to take an active role in research on ageing and health are scarce and thus warranted [5]. Among the few published studies is a survey investigating the interest in public involvement in research among students at a senior university in Switzerland [27]. In this study, higher age and higher level of education corresponded with the likelihood of having previously been actively involved in research. When examining future interest in public involvement in research, 62% of the population stated that they had such an interest, and the likelihood was higher for those with a higher age, higher level of education, and previous experience. Another study based on survey data from close to 6,000 respondents in six European countries explored the willingness of people–not limited to older people–to be actively involved in life sciences research and investigated incentives that motivate such involvement [28]. The response rate for each country was only 5%, but the results showed that 66.8% of the respondents were willing to be actively involved in life science research projects.

Summing up, the importance of the active involvement of older people in research is ethically and epistemically motivated and has firm political support [13]. However, more systematic, and comprehensive knowledge of awareness, attitudes, and willingness in relation to public involvement in research is needed to be able to understand the challenges researchers face when seeking to foster participatory approaches in research.

### Aim and research questions

The overarching aim of the present study was to describe the initial findings from a panel study on user involvement in research on ageing and health targeting people aged 60 years and older in Sweden and to investigate their awareness and attitudes towards public involvement in research. The specific research questions were: 1) What are the awareness, attitudes and willingness towards public involvement in research among older people in Sweden? 2) How does the willingness of older people in Sweden to be actively involved in research on ageing and health relate to demographics and self-rated health? Moreover, is this relationship influenced by previous active involvement in research?

## Materials and methods

This study was based on data collected within a national longitudinal panel study [5], which is part of the UserAge research program [3], engaging researchers at four universities in Southern Sweden as well as user representatives. The panel study focuses on user involvement in research, addressing four different samples [5]. The present study was based on data collected in the first data collection wave administered in 2019–2020, targeting the population aged 60 years and older in Sweden.

### Respondents and recruitment

Based on population data from Statistics Sweden [29] there are approximately 2.66 million (53% women) people aged 60 years and older in Sweden. Using a confidence level of 95% and a margin of error of 4, we estimated a total sample size of 1,200 to be representative [30] for the total 60+ population of Sweden. Based on previous experience [31] and a pilot study, the expected response rate was estimated to 50–60%. Accordingly, an invitation to participate was sent out to 3,427 individuals 60 years and older, randomly selected from the Swedish national population registry.

### Survey variables

The survey included 27 questions divided into the following sections: Awareness of and previous experience of public involvement in research (Q1-4); Attitudes towards public involvement in research (Q5); Interest in research and willingness to be actively involved in research (Q6-8); Facilitators and barriers for public involvement in research (Q9-13); Demographic questions (Q14-22); Self-rated health and frailty (Q23-27). In the present study, data from all survey questions, except for a few demographic variables and those on facilitators and barriers, were used.

### Demographics

The demographic variables used were age, sex, level of education, and employment status. Level of education was categorized as Elementary school, Upper Secondary School, College or University less than 3 years, and College or University 3 years or more. Employment status was used as a dichotomized variable to the survey question, “What is your main occupation right now?”, with “retired” assigned to one option and the other alternatives (Employed; Self-employed; Student; Looking for work; Long-term sick leave (more than 3 months)”; Other, namely…) assigned to the other.

The respondents’ financial situation was captured by the question, “How would you best describe your financial situation?”, with a Likert scale consisting of five response alternatives ranging from poor to excellent. Place of residence was based on the classification produced by the Swedish Association of Local Authorities and Regions (SALAR) [32] in which municipalities are divided into three broad categories (Large cities, Medium-sized towns, and Smaller towns/rural areas/rural municipalities) based on structural parameters such as population and commuting patterns.

### Self-rated health and risk of frailty

The question, “In general would you say your health is…?” based on the SF-36 questionnaire [33] was used for self-rated health, with a Likert response scale consisting of five response alternatives ranging from poor to excellent. Risk of frailty entails weakness, reduced physical activity, and impaired balance [34]. Four questions were used as crude early potential indicators of frailty: “Can you walk for about 15–20 minutes?”; “Have you felt generally tired or experienced reduced strength in the last three months?”; “Do you fall often or are you ever afraid of falling?”; “Do you need help with shopping (i.e. getting to the store, picking out groceries, paying, and carrying groceries home)?” [35]. We considered a respondent to be at risk of frailty if he/she fulfilled two or more of the frailty indicators [35].

### Awareness and previous experience

Awareness of public involvement in research on ageing and health was captured with the question “Are you aware that you as an individual can take an active part in the research process?”, with three response options: Yes; No and Maybe. These response options were dichotomized into Yes (Yes + Maybe) and No when testing how awareness related to respondent characteristics.

Any previous active involvement in research was determined by a multiple-choice question where the respondents were asked to tick the response options to indicate in what way they had been actively involved in the past. They were also given the opportunity to give an open-ended response in free text. For the open-ended responses, a manual assessment was made by several researchers in consultation on whether the answers given constituted an active involvement in research or not. Based on the assessment of whether the respondents had any form of active involvement in the past, the dichotomous variable “Previous active involvement” was created, and the response options were categorized appropriately.

### Attitudes and willingness

Attitudes towards public involvement in research on ageing and health were captured through statements rated with a Likert response scale, ranging from strongly agree (1) to strongly disagree (5). Statements such as, “Involving citizens actively in research: …can enhance research communication”, or “…means that the objectivity, independence, and integrity of the research are jeopardized” were presented.

Attitudes were further explored through questions related to respondents’ preferred form of involvement in research. The respondents were asked to take a position on the statement, “If you were given the opportunity, how likely is it that you would like to participate by…” followed by six examples of active involvement, such as “Being part of a user council, reference group, board or the like” and an open-ended response option. A Likert response scale was used, ranging from very much (5) to not at all (1).

Willingness to be actively involved in research on ageing and health was captured with the question, “Would you consider being actively involved in research on ageing and health?” with the response options Yes, No and Maybe, dichotomized into Yes (Yes + Maybe) and No, when investigating how the respondents’ characteristics were related to willingness.

### Procedure

In spring 2019, the authors drafted a set of survey questions about awareness of and attitudes towards public involvement in research. The survey questionnaire was constructed based on existing literature and input from the UserAge research team. Next, a user forum consisting of eight persons aged 60 years and older (recruited through snowballing within the network of the Centre for Ageing and Supportive Environments (CASE) at Lund University) and three researchers was formed [36]. During three sessions, the user forum refined the survey for readability and time/length to complete. A professional survey company was then commissioned for the practical implementation of the survey, which opened in late August 2019 and closed in November 2019. The potential respondents received an invitation letter by post including instructions on how to complete the survey online, on paper, or by telephone. About two weeks later, those who did not complete the survey online were contacted by phone by the survey company and reminded about the three options to complete the survey.

To assess the quality of data collection, one researcher (OJ) listened-in on 5% of all telephone surveys. After 10% of the surveys had been answered, a quality check of the data that had been collected so far was carried out by the survey company.

### Data analysis

To describe awareness of, attitudes towards, and willingness to participate in research, standard descriptive analyses were used. There was a variation in sample size across the analysis due to missing data for certain items.

To test how awareness and previous experience of public involvement in research related to respondent characteristics (demographics, self-rated health, and attitudes), the Chi-square test (χ^2^) and Mann Whitney tests were used. The Chi-square test (χ^2^) was used in relation to variables that had a nominal scale (Sex, Employment status, Born in Sweden, Risk of frailty, Place of residence), and the Mann Whitney test was used for variables that had either a continuous (Age) or an ordinal scale (Level of education, Financial situation, Self-rated health).

An initial logistic regression model was set up including the independent variables Sex, Employment status, Born in Sweden, Risk of frailty, Place of residence, Age, Level of education, Financial situation, and Self-rated health, to investigate how they were related to willingness to be actively involved in research on ageing and health (dependent variable). Before the model was set up, multicollinearity between the independent variables was checked using Pearson Correlation. Using the same independent variables in a second model, the variable “Previous active involvement” was used as an additional independent variable.

We used IBM SPSS software versions 26 and 27 [37] for all analyses. A two‐sided p-value of <0.05 and a 95% confidence interval (CI) served as indicators of statistical significance.

### Ethical considerations

Ethical approval was obtained from the Ethical Review Board in Lund (No. 2018/986). The postal invitation letter that was sent by post to all potential respondents included information on the background and purpose of the panel study, informed that participation was voluntary, that respondents had the right to discontinue their participation at any time, and that data would be handled in accordance with the General Data Protection Regulation (GDPR) and local data protection guidelines. In addition, verbal information was provided by the survey company staff, who specialized in conducting telephone interviews and had undergone project-specific training. Each respondent provided informed consent either by completing the survey online or on paper. The verbal informed consents were documented by the telephone interviewers. The data were encrypted and stored by the survey company in a secure database, then transferred to the researchers and stored in a high-security platform (LUSEC) at the Faculty of Medicine, Lund University. Only project researchers had access to the data.

## Results

### Response rate

In total, 3,427 persons were informed about the study and asked to participate, and 881 completed the survey (26% response rate): 41% (n = 361) online, 32% (n = 282) on paper, and 27% (n = 238) via telephone.

### Descriptive baseline results

The respondents were 60–97 years old with a mean age of 72.2 (SD = 7.30) years. The sex distribution in the sample was 52.9% (n = 462) women and 47.1% (n = 412) men. In terms of education, 24.2% (n = 210) stated elementary school as their highest level of education, 17.9% (n = 155) upper secondary school, 23.2% (n = 201) college less than three years, and 34.8% (n = 302) college three years or more. When it comes to self-rated health, 38.5% (n = 338) reported their health as good, 23.9% (n = 210) as very good, and 10.4% (n = 91) as excellent, while 24.6% (n = 216) reported their health as fair and 2.5% (n = 22) as poor. For more details about the baseline results, see Table 1.

**Table 1:** Respondent characteristics and characteristics related to respondents’ awareness of the possibility to be actively involved in research on ageing and previous experience of being actively involved in research.

Characteristic	Total %^1^ (*n*)	Awareness^2^ (*n* = 879)		P*-*value^3, 4^	Previous experience^2^ (*n* = 864)		P*-*value^3, 4^
		Yes/Uncertain % (*n*)	No % (*n*)		Yes % (*n*)	No % (*n*)	
*Age*, mean (SD)	72.2 (7.30)	72.0 (7.29)	72.3 (7.32)	0.614	72.1 (7.95)	72.2 (7.20)	0.460
60–69 years	40.3 (352)						
70–79 years	43.6 (381)						
80–89 years	14.1 (123)						
≥ 90 years	2.1 (18)						
*Sex* (n = 874)				0.204			0.136
Men	47.1 (412)	41.2 (169)	58.8 (241)		15.4 (62)	84.6 (341)	
Women	52.9 (462)	37.0 (171)	63.0 (291)		11.9 (54)	88.1 (400)	
*Level of education* (n = 868)				**< 0.001**			**< 0.001**
Elementary school	24.2 (210)	28.2 (59)	71.8 (150)		6.7 (14)	93.3 (195)	
Upper secondary school	17.9 (155)	39.6 (61)	60.4 (93)		8.1 (12)	91.9 (137)	
College less than 3 yrs	23.2 (201)	39.3 (79)	60.7 (122)		13.3 (26)	86.7 (170)	
College 3 yrs or more	34.8 (302)	45.7 (138)	54.3 (164)		21.5 (64)	78.5 (233)	
*Employment status* (n = 866)				0.140			0.146
Not retired	20.6 (178)	44.1 (78)	55.9 (99)		17.1 (30)	82.9 (145)	
Retired	79.4 (688)	38.0 (261)	62.0 (426)		12.9 (87)	87.1 (588)	
*Born in Sweden* (n = 877)				0.918			0.821
No	8.9 (78)	39.7 (31)	60.3 (47)		13.0 (10)	87.0 (67)	
Yes	91.1 (799)	39.1 (312)	60.9 (485)		13.9 (109)	86.1 (674)	
*Financial situation* (n = 869)				0.653			0.052
Poor	5.3 (46)	45.7 (21)	54.3 (25)		9.1 (4)	90.9 (40)	
Fair	26.0 (226)	39.1 (88)	60.9 (137)		11.7 (26)	88.3 (197)	
Good	44.9 (390)	36.4 (142)	63.6 (248)		13.8 (53)	86.2 (331)	
Very good	17.7 (154)	43.8 (67)	56.2 (86)		16.7 (25)	83.3 (125)	
Excellent	6.1 (53)	43.4 (23)	56.6 (30)		19.2 (10)	80.8 (42)	
*Self-rated health* (n = 877)				0.978			0.381
Poor	2.5 (22)	63.6 (14)	36.4 (8)		18.2 (4)	81.8 (18)	
Fair	24.6 (216)	35.8 (77)	64.2 (138)		12.3 (26)	87.7 (186)	
Good	38.5 (338)	39.1 (132)	60.9 (206)		13.4 (44)	86.6 (285)	
Very good	23.9 (210)	41.1 (86)	58.9 (123)		14.0 (29)	86.0 (178)	
Excellent	10.4 (91)	36.3 (33)	63.7 (58)		17.8 (16)	82.2 (74)	
*Risk of frailty* (n = 866)				0.758			0.358
No	88.9 (770)	39.5 (304)	60.5 (465)		13.4 (101)	86.6 (653)	
Yes	11.1 (96)	37.9 (36)	62.1 (59)		16.8 (16)	83.2 (79)	
*Place of residence* (n = 874)				0.091			0.748
Large city	30.7 (268)	43.3 (116)	56.7 (152)		14.8 (39)	85.2 (224)	
Medium-sized town	39.8 (348)	39.4 (137)	60.6 (211)		13.2 (45)	86.8 (296)	
Smaller town/rural area	29.5 (258)	34.0 (87)	66.0 (169)		12.6 (32)	87.4 (221)	

^1^ Valid percent

^2^ *n* varies due to internal missing

^3^ Chi-square test was used in relation to variables that had a nominal scale (Sex, Employment status, Born in Sweden, Risk of frailty, Place of residence) and Mann Whitney U tests for variables that had either a continuous (Age) or an ordinal scale (Level of education, Financial situation, Self-rated health).

^4^ Statistically significant *p*-values are bolded.

### Awareness, previous experience, and attitudes

Slightly more than one third 39.0% (n = 343), responded that they were aware that they as members of the public could be actively involved in research, 16.2% (n = 142) that they were uncertain and 44.8% (n = 394) that they were not aware. When the response options were dichotomized [(Yes + Uncertain) vs. No], we found a statistically significant difference between awareness of public involvement in research and level of education, with a higher level of education being associated with a higher level of awareness (Table 1).

A low share (13.8%; n = 119) replied that they had previous experience of being actively involved in research in at least one way. While only slightly higher than other forms of involvement, participating in communicating research results was the most common form of active involvement (5.9%; n = 50). A similar proportion of respondents (5.2%; n = 45) had experience of participating in a user board, reference group/council or similar, had had a consultant role in research, or had conducted interviews or made measurements and communicated them for use in research.

We found a statistically significant association between previous experience of public involvement in research and level of education, with a higher level of education being associated with an increased probability of having previous experience of being involved in research (Table 1).

### Attitudes and willingness to participate

**Fig 1:**
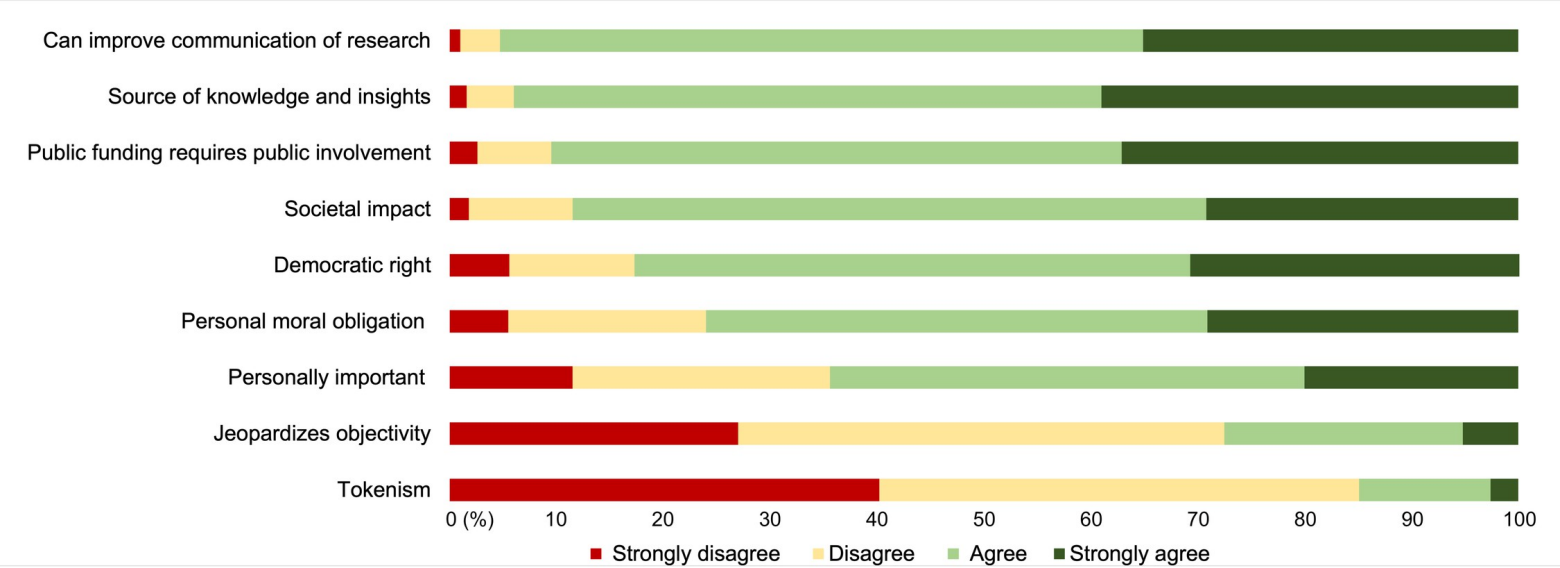
Attitudes towards public involvement in research on ageing and health.

Regarding attitudes towards public involvement in research on ageing and health (Fig 1), close to all respondents (95.3%; n = 776) agreed with the statement that public involvement in research could improve the communication and outreach of research results. A similar proportion of respondents (94.0%; n = 805) thought that they should be actively involved because they could contribute valuable knowledge, insights, and experiences on ageing (Fig 1). Furthermore, 90.5% (n = 778) agreed that it was important for citizens to be actively involved in research on ageing and health, especially research that was publicly funded. The two statements that the respondents felt were the least in line with their views were that active engagement of citizens in research risks jeopardizing the objectivity, independence, and integrity of the research (27.5%; n = 227), and the statement that public involvement was only a form of tokenism and had no bearing on the results (14.9%; n = 122).

Regarding willingness to be actively involved, when asked the question, “Would you consider being actively involved in research on ageing and health?”, 41.4% (n = 357) responded that it was something they would consider, 27.1% (n = 234) that they might consider it, and 31.5% (n = 272) responded that it was something they would not consider.

**Fig 2:**
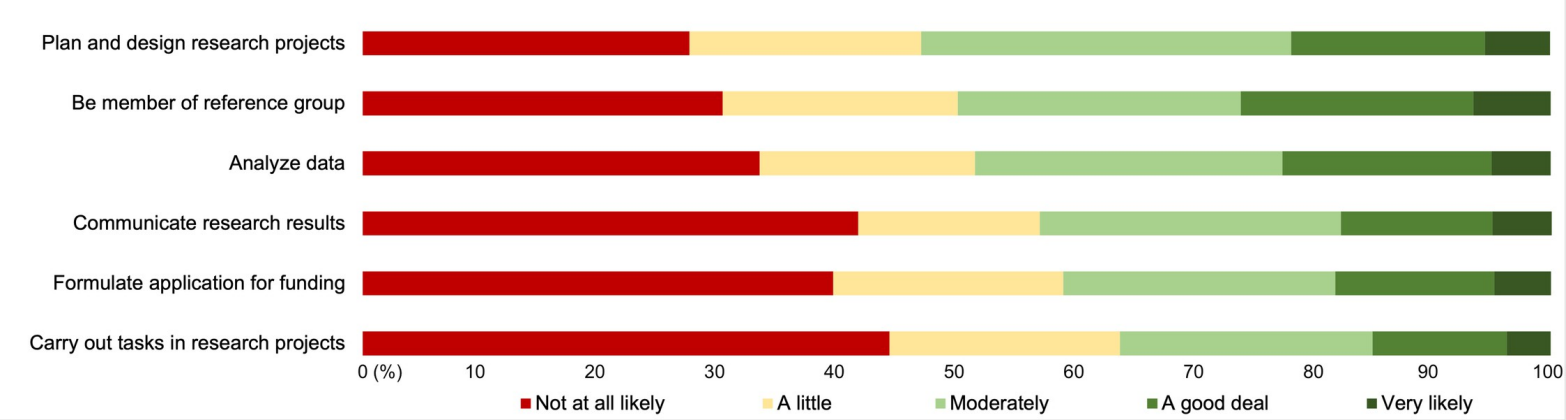
Willingness to be actively involved in research on ageing and health through various means.

Among the respondents who stated that they would or might consider being actively involved in research (68.5%; n = 591), the attitudes were most positive towards contributing to the planning and design of research projects (Fig 2). Here, a slight majority (53.0%; n = 318) stated that they were moderately to very likely to be involved if given the opportunity. A close second was being a member of a user board, reference group/council or similar where the corresponding figure was 49.9% (n = 300). The respondents were least positive towards carrying out tasks (e.g., recruit participants, collect data, etc.) in research projects where 36.3% (n = 215) stated they were moderately to very likely to be involved.

### Willingness in relation to demographics and health

A higher level of education was associated with higher odds of being willing to be actively involved in research (Table 2). That is, an individual with a college degree of 3 years or more had about 3.16 (95% CI, 2.05–4.87) times higher odds of being willing to be actively involved in research compared to a person whose highest level of education was elementary school. For an individual whose highest level of education was the completion of upper secondary school, the corresponding ratio (OR) was 2.56 (95% CI, 1.56–4.18), and for an individual with a degree from college of less than 3 years, the ratio was 1.67 (95% CI, 1.08–2.57).

**Table 2:** Logistic regression results for willingness to be actively involved in research.

Independent variable	Model 1^1, 2^ (n = 815)		Model 2^1, 2, 3^ (n = 799)	
	Odds Ratio (95% CI)	P-value^4^	Odds Ratio (95% CI)	P-value^4^
*Age*	0.977 (0.953–1.002)	0.074	.975 (.950–1.001)	0.058
*Sex*				
Men	Ref.		Ref.	
Women	0.926 (0.672–1.275)	0.636	.964 (0.695–1.336)	0.825
*Level of education*				
Elementary school	Ref.		Ref.	
Upper secondary school	2.551 (1.559–4.176)	**<0.001**	2.419 (1.466–3.992)	**<0.001**
College less than 3 years	1.664 (1.077–2.568)	**0.022**	1.481 (0.950–2.308)	0.083
College 3 years or more	3.156 (2.047–4.866)	**<0.001**	2.648 (1.703–4.116)	**<0.001**
*Employment status*				
Not retired	Ref.		Ref.	
Retired	0.730 (0.453–1.177)	0.197	0.761 (.466–1.242)	0.274
*Born in Sweden*				
Yes	Ref.		Ref.	
No	.687 (0.397–1.189)	0.180	0.751 (0.431–1.310)	0.313
*Financial situation:*				
Poor-Fair	Ref.		Ref.	
Good	1.647 (1.132–2.395)	**0.009**	1.623 (1.105–2.385)	**0.014**
Very good-Excellent	1.101 (0.700–1.733)	0.678	1.070 (0.672–1.704)	0.775
*Self-rated health:*				
Poor-Fair	Ref.		Ref.	
Good	0.782 (0.513–1.191)	0.251	0.719 (0.464–1.112)	0.138
Very good-Excellent	1.151 (0.718–1.843)	0.559	1.094 (0.674–1.777)	0.716
*Risk of frailty*				
No	Ref.		Ref.	
Yes	0.754 (0.444–1.282)	0.297	0.695 (0.401–1.202)	0.193
*Municipality classification*				
Larger city	Ref.		Ref.	
Medium-sized town	1.080 (0.738–1.580)	0.691	1.106 (0.750–1.632)	0.611
Smaller town/rural area	1.035 (0.686–1.563)	0.870	1.077 (0.707–1.639)	0.731
*Confounder*				
Previous experience			5.436 (2.634–11.219)	**<0.001**

^1^ Overall model fit: Model 1: -2 log likelihood = 934.083; Model 2: -2 log likelihood = 102.486.

^2^ n varies due to internal missing

^3^ In Model 2, previous experience of active involvement in research was included as a confounder.

^4^ Statistically significant *p*-values are bolded.

Persons who rated their financial situation as Good had on average 1.65 (95% CI, 1.13–2.40) times higher odds of being willing to be actively involved in research compared to a person who rated his or her financial situation as Poor-Fair. However, there was no statistically significant increased odds for an individual who rated his or her financial situation as Very good-Excellent compared to a person who rated his or her financial situation as Poor-Fair.

Furthermore, persons who had previous experience of being actively involved had on average 5.44 (95% CI, 2.64–11.22) times higher odds to be willing to be actively involved again. After adjusting for previous experience of being actively involved in research (Table 2), college of less than 3 years was no longer statistically significant in the adjusted model (OR = 1.48, 95% CI, 0.95–2.31, p = 0.083).

## Discussion

The main results from this study are that about four out of ten respondents were aware that they could be actively involved in research, and that the odds of being willing to be actively involved were significantly higher for those with previous experience and a higher level of education. An important result in its own right, the low response rate indicates that it is challenging to generate interest for “research about research” among the general public. While the response rate was low, other survey studies show similar or remarkably lower response rates [28, 38]. Still, when interpreting the survey results, it is important to keep the relatively low response rate in mind and consider the potential implications. In the analyses of traditional study participation in randomized controlled “digital health technology” studies, Poli et al. [39] recently demonstrated that the participation of older people is selective by age, sex, health status, job level, and digital skills. Our study carries a similar risk for bias.

### Demographics and self-rated health

What most clearly distinguishes the study sample from Sweden’s population of people 60 years or older is the high level of education. That is, 58% of the study sample had a post-secondary education compared to 17% in the overall 60+ population [29]. However, it should also be pointed out that our 60+ sample contains a somewhat lower proportion of people 80 years and older (16%) compared to Sweden’s 80+ population (20%), which may have some relevance as the level of education is generally lower in this age group in a Swedish context [29].

When it comes to self-rated health, 34% (n = 301) reported their health as excellent or very good. This can be compared to a cross-sectional population-based sample of 70-year-olds from a Swedish cohort study (n = 1,136) where 50% of men and 47% of women reported their health as excellent or very good [40]. It can also be compared to a study of very old people (M age = 85.1; SD = 2.95) in Sweden (n = 397) where 72% rated their health as at least good [41], compared to 73% in our sample. Although increasing age does not always result in a decline in self-rated health as older people are often able to adapt to poorer health [42], these comparisons still give the impression that the ratings of health in our sample were somewhat low for the age group studied.

### Willingness to be actively involved in research

The respondents in the survey reported a high willingness to be actively involved in research, since 68.5% expressed that this was something they would or might consider doing. This can be compared with two earlier studies. In the study from Switzerland, which was directed at students at a senior university, 62% stated they were interested in research projects with public involvement [27]. In the second study, which was directed at 6,000 respondents in six European countries–and not limited to older people– 67% answered that they were willing to be actively involved in research in life sciences [28]. In other words, the proportions are relatively similar even though the samples in the few previous larger studies were different.

### Awareness and attitudes towards public involvement in research

About four out of ten respondents were aware of the possibility of being actively involved in research, and there are no previous studies that we could find that explored the awareness of this specific category of users. The respondents considered public involvement important mainly because it could improve the communication and outreach of research results, but also because it is a way that they can contribute their valuable experiences regarding ageing. The fact that older people so clearly and explicitly emphasize that their willingness to be actively involved in research is rooted in the desire to contribute to quality improvements is in accordance with previous research [18]. However, the driving force found in Fudge et al.’s study was about achieving a real change in practice, and not just to facilitate or enrich the research process. Still, our study gives a clear indication that the motivation to make a difference constitutes a central driving force for active involvement in research on ageing and health. Regarding attitudes towards active involvement in research among frail older people in Sweden, Berge et al.’s qualitative study indicates that their interest in and ability to be actively involved in research is highly varied and individual, which requires a great deal of sensitivity in each individual case [20]. In our sample, 11.1% (n = 96) were categorized as in risk of frailty, but the variable was not associated with either awareness, previous experience, or willingness to participate. This supports Berge et al.’s conclusion that the interest in active involvement in research varies among frail older people.

### How previous experience relates to public involvement in research

When it comes to previous experience of active involvement in research, 14% is a relatively low figure compared to the results of Seifert et al. [27], where the corresponding proportion was 24%. However, given that their study targeted older people at a senior university and not older people in the general population, this is not surprising. Moreover, the significant association between previous involvement in research and willingness to be involved in research again in our study is in line with the results of the aforementioned study from Switzerland [27]. Thus, it seems as if the experience of having been actively involved in research corresponds to a higher willingness to get involved in research again.

Although a clear majority of respondents expressed a positive attitude towards public involvement in research, it also emerged that a non-negligible proportion (1/7) regarded public involvement as a form of tokenism. This is in line with previous research among older people [25]. However, a closer look at this sub-sample shows that those who harbour this perception and also have previous experience of actively participating in research only amounted to 1.8% (n = 13) of the total sample. In other words, the notion that public involvement in research is a form of tokenism does not seem to be based heavily on actual personal experiences. The reasons for such attitudes remain to be explored.

### How education relates to public involvement in research

Previous active involvement was not the only factor that affected willingness to participate. For respondents with a higher level of education, the probability of being willing to be actively involved in research was significantly higher, and this applied regardless of whether respondents had been involved in participatory research approaches before. There was one exception: among respondents with a college degree equivalent to less than three years of study, the probability was lower. In addition, a higher level of education was associated with an increased possibility that respondents were aware of the possibility to be actively involved in research, as well as with previous active involvement in research. A plausible explanation is that the association between willingness to participate and higher levels of education relates to linguistic ability. Familiarity with academic language is something that develops during schooling and could constitute a facilitator for approaching academic discourse. On the other hand, lack of familiarity could present an obstacle. This is something that is supported by linguist Michael Halliday’s theory that learning consists of the development of ‘registers’, which move from the more concrete ‘everyday language’ to the more abstract ‘language of education’ [43]. Thus, the lack of sufficient academic registers could very well be a plausible explanation for this result (see [44] for a more elaborate discussion on this). The development of academic registers relates to Archer et al.’s theory of ‘science capital’, which in addition to knowledge includes attitudes, behaviours, and social contacts and networks [45]. It would be interesting and important to investigate this further in future studies.

### How age relates to willingness to be actively involved in research

The fact that age emerged as statistically significant after adjustment for previous experience, with increased age affecting willingness negatively, is an interesting result, not least as Seifert et al. [27] indicated an inverse relationship. Once again, it should be noted that their study sample only included people at a senior university. Congruent with our results, a recently published study of attitudes towards active involvement in research among informal carers in Sweden [46] indicates that older age is significantly associated with less interest in being actively involved in research. One presumable reason is that deteriorating health comes with increasing age, and this result may be a matter of ability. That health conditions can negatively affect the willingness to be actively involved in research has been indicated by several other studies [19–22, 39]. What speaks against this reasoning, however, is that in our material, ‘self-rated health’ did not correlate with age, and there was no association between ‘self-rated health’ and willingness to be actively involved in research. Accordingly, these complex dynamics warrant further investigation.

### Strengths and limitations

As research on public involvement in research so far has been dominated by small-scale, often retrospective, qualitative studies, this quantitative study with a substantial sample size makes a valuable contribution that will serve as the basis for further study.

Although the survey was developed in collaboration between researchers and representatives of the target group, comments in free-text answers and experiences from listening-in on telephone interviews indicated that the concept of ‘active involvement’, as well as the questions asked, were sometimes considered abstract and difficult to understand. This indicates a risk that the survey was perceived as intimidating by some respondents [47]. Furthermore, it highlights the difficulties of making academic language sufficiently accessible to people outside of academia.

The potential respondents were given three options to complete the survey. Even though the online response option was the most used, the distribution between online, paper, and telephone responses was relatively even. While the response rate was nevertheless low, the fact that respondents were offered several ways to respond might have increased the response rate compared to if they had only been offered one response mode [48].

Moreover, we chose to target the general public and not specifically people who are active in interest groups–even if such individuals were included in the study sample. Whether there are differences between individuals in the general population and individuals who represent particular interest groups warrants further study. Similarly, further research is needed to find out to what extent the methodology we used can be employed in other contexts, as well as to investigate to what extent our results regarding the population’s awareness, attitudes, and willingness to participate are valid elsewhere.

## Conclusions

By responding to the lack of research, and in particular the lack of larger quantitative studies, this study contributes with knowledge about awareness of and attitudes towards public involvement in research among people aged 60 years or older in Sweden. The study’s low response rate shows that it is a challenging task to motivate older people in Sweden to participate in such research. This is especially true for people with lower education who were substantially underrepresented in our sample. The predominantly positive but still varying attitudes towards different forms of public involvement in research among the respondents indicate that it is important to design research projects with a sensitivity to older people’s interests and conditions. The fact that less than half of the respondents were aware of the possibility to be actively involved in research indicates that there is potential to reach out to substantial numbers of older people for whom public involvement in research is not known. Such efforts are ultimately a question of enhancing democracy, as well as a question of striving to ensure that important knowledge and essential perspectives are not neglected in research on ageing and health.

As to future research, more research is warranted to explore how an increased awareness of and willingness to be involved in research can be achieved in particular among people with lower education. Examples of additional research questions are why certain older people seem to regard public involvement as a form of tokenism, and how linguistic ability and ‘science capital’ relate to willingness to get involved in research.
